# The Mix Ratio Study of Self-Stressed Anti-Washout Underwater Concrete Used in Nondrainage Strengthening

**DOI:** 10.3390/ma12020324

**Published:** 2019-01-21

**Authors:** Shaofeng Wu, Shao-Fei Jiang, Sheng Shen, Zhaoqi Wu

**Affiliations:** College of Civil Engineering, Fuzhou University, Fuzhou 350108, China; shaofeng45@163.com (S.W.); s_shen@fzu.edu.cn (S.S.); zhaoqi_wu@163.com (Z.W.)

**Keywords:** nondrainage strengthening, self-stressed anti-washout underwater concrete (SSAWC), mix ratio, orthogonal test design, optimization

## Abstract

Anti-washout underwater concrete (AWC) is widely used in nondrainage strengthening; however, there still exist some problems with it, such as high strength loss and poor interfacial bond in practical engineering application. Based on the study of self-stressed concrete (SSC), a research on the mix ratio for the C30 self-stressed anti-washout underwater concrete (SSAWC) was carried out in this paper in hope of solving the above problems, specifically, by adding an expansive agent to the AWC. The parameters, such as strength, fluidity, anti-dispersity, and expansibility, were picked as target indices in determination of the mix ratio. The orthogonal test design and range analysis were used to determine the reasonable mix ratio and study the influence of various parameters on the performance of SSAWC. The experimental program conducted includes a series of strength, fluidity, anti-dispersity, and expansibility tests on 18 groups of specimens. The results show that C30 SSAWC has an excellent performance using the optimal mix ratio. Compared with AWC, the expansibility and self-stress of the SSAWC can be easily observed, and the compressive strength ratio of the SSAWC casted in water to that casted in air is much bigger. This implies that SSAWC is applicable to the nondrainage strengthening.

## 1. Introduction

In recent years, a growing number of in-service bridges need strengthening out of concern for both structural safety and smooth transportation [1]. It has been found by the maintenance engineers that the strengthening of substructures is more difficult than that of superstructures because the substructures of a bridge include structures that lie under water most of the year, e.g., piles, piers, etc. Conventional strengthening methods for the substructure, such as enlarging section method [2], bonded steel plates method [3], fiber-reinforced polymer (FRP) method [4,5,6], extraneous prestressed strengthening technique [7], and other novel methods [8,9], do improve the load capacity and durability of substructure. However, they are more often than not expensive, time-consuming, and traffic-disrupting, because cofferdam needs to be built for drainage first before any work could be done to underwater substructures. Therefore, alternative strengthening methods that make drainage no longer necessary, such as jacket strengthening method [10], FRP underwater strengthening method [11,12], and precast concrete segment assembly method [13], have been proposed in an effort to solve the above-mentioned problems caused by drainage. These strengthening methods are economical, fast, and traffic friendly compared with conventional methods. But all these methods require anti-washout underwater concrete (AWC) [14], also known as non-dispersible underwater concrete (NDC) [15,16], to be casted in-between the original concrete and the repair material, where a weak bond is supposed to exist between these layers, making it a vulnerable spot in the structure. 

AWC refers to the concrete mixed up with anti-washout admixture (AWA, also called an anti-dispersion agent). It can be poured under water and its aggregate and cement slurry are far less likely to separate in water than that of Portland cement. The AWA is a kind of water-soluble polymers with long-chain structures and strong absorption capacity, and there are two kinds of AWA for AWC: acroleic acid series and fiber series [17]. Owing to some remarkable advantages, such as good anti-dispersibility, self-leveling ability, and nonpollution, as shown in practical applications, AWC has attracted increasing attention from scholars and engineers in the research areas of material performance and mix ratio design. Problems concerning AWC have been discussed with possible solutions proposed. 

The first concern is about strength, one of the most important performance indices for AWC. However, the performance could be compromised due to the loss of cement and fine particles as well as the segregation of coarse aggregates upon casting in water [18]. Heniegal expressed the reduction in strength as cube compressive strength ratio (*t*) of specimens made underwater to those made in air [19]. In his experiments, strength ratio *t* increased from less than 0.4 to more than 0.9 by increasing AWA and cement contents. Therefore, it could be safely inferred that the increase of AWA and cement contents would result in lower strength reduction. 

The second concern is about fluidity, one of the important indices for AWC, generally evaluated by slump and slump flow [20]. The flocculation produced by AWA could impair the fluidity and thus result in fluidity loss over time faster than common concrete. Niu [21] reported that the slump flow of AWC was more than 500 mm, and no loss was witnessed in 3 h by replacing 30% of cement with fly ash. In Zhang’s experiments [22], the slump flow of AWC achieved the maximum (473 mm) when the sand rate (the ratio of the quality of fine aggregate to the quality of all of aggregate) was limited to a certain range (42%). Consequently, it is possible to increase the fluidity of AWC by adding fly ash, or adjusting the sand rate. 

The third concern is about anti-dispersity. Anti-dispersity is another important index for AWC, dependent on the amount and type of AWAs. Zhang [23] found that the increasing content of AWAs brought about not only an increase in anti-dispersity, but also a decrease in fluidity. Thus, the content of AWAs needs to be limited to a certain extent. For example, the appropriate content of AWA with a molecular weight of 10 million is 8–10%. In addition, Kamal [24] found that the anti-dispersity could increase more than 20% by replacing 8% cement with silica fume. These experimental results indicate that anti-dispersity of AWC can be improved by increasing the AWA and silica fume content.

The fourth concern is about durability. Durability is also a significant reflection of the characteristic of concrete, usually evaluated by the impermeability of water and chloride [25,26,27]. Naik [28] reported that the permeability of water and chloride is reduced by more than 50% when 50% cement is replaced with fly ash in AWC. Furthermore, Zhao [29] found the Chloride ion permeation coefficient is reduced by 20% when 0.7% water-proofing is added to AWC. Hence, adding fly ash or water-proofing agent can reduce the permeability of AWC.

As mentioned above, most of the researches have been focused on the strength, fluidity, anti-dispersibility, and durability of AWC, and few researchers pay attention to the effect of expansion for AWC. The importance of AWC expansion in strengthening without drainage is manifest in two aspects: (1) alleviating shrinkage in the curing of AWC and improving the bond strength of AWC and (2) making the optimum use of high strength of AWC by applying prestress. Although people believe that the shrinkage of AWC is partly restricted due to underwater curing [30], it is significant to eliminate the shrinkage of AWC as much as possible because the shrinkage can reduce the bond strength between the AWC and its adjacent members. A suitable solution is to add expansive agent in AWC. Qu and Liu [31] compared some steel tubes filled with expansive concrete with those filled with normal concrete. The results showed that the bond strength between the expansive concrete (dosage of expansive agent being 10%) and the cold-formed steel tube (200 × 250 × 10 × 600 mm) was almost 1.5 times (1.32 MPa) more than that between the normal concrete and the steel tube (0.83 MPa). Lu [32] investigated the bond behavior of steel tube columns filled with self-stressing and self-compacting concrete. Experimental results showed that the bond strength of specimens varies from 0.50 MPa to 2.51 MPa. Self-stress significantly increases the bond strength of specimens, with an average increment of 42.7%. Therefore, it could be concluded that the expansion of concrete can significantly improve the bond strength. In addition, the long-term underwater curing is helpful to the bond strength development of AWC. In the experiments carried out by García Calvo [33], the expansion of specimens via a 28d underwater curing at 20 ± 2 °C was more than 0.15%, while that value of specimens cured in the air during the same time and temperature was less than −0.01%. Furthermore, the status of “triaxial compression” can be produced by placing a steel or FRP tube outside the expanding AWC. This may increase the ultimate strength of AWC remarkably. In the experiments carried out by Qu and Liu [31], the ultimate cube compressive strength of the specimen with 10% expansive agent increased by 60% compared with that of the normal concrete specimen. Therefore, adding proper amount of expansive agent to AWC could be an effective way to solve the problem of bond strength between the AWC and the adjacent member in nondrainage strengthening. 

In summary, the purpose of this paper is to develop an improved AWC, i.e., the self-stressed and anti-washout underwater concrete (SSAWC) as we called it, which not only enjoys high bond strength, but also produces a “triaxial compression” for the concrete core of compressive members. Meanwhile, the SSAWC is expected to satisfy the requirements of strength, fluidity, anti-dispersity, and durability. In this paper, the development of C30 SSAWC is taken as an example of the proposed method, and SSAWC of other strength grade can also be prepared as such. 

## 2. Experimental Procedures

### 2.1. Performance Requirements

Generally, the performance indices for SSAWC should be determined according to the requirements of both construction and strengthening. Based on the above-mentioned in Section 1, four indices are considered in this experiment: fluidity, anti-dispersity, strength, and expansibility. Durability is not considered in this study simply because the test of durability usually takes a great length of time. The performance requirements of SSAWC are shown as follows.

(1) Fluidity is usually evaluated by slump and slump flow at 2 min following removal of the cone. Because it is difficult to vibrate the poured SSAWC in the pouring process, the concrete has to be endued with enough uniformity and self-compactness. Therefore, the slump and slump flow of the SSAWC are expected to reach 230 ± 30 mm and 450 ± 30 mm, respectively, according to the code [34].

(2) Anti-dispersity endows the SSAWC with less flowability, good cohesive property, and water retention. The pH method was applied to evaluate anti-dispersity. When the pH is close to 7, it results in better anti-dispersity. The pH value of SSAWC should be less than 12 according to the code [34].

(3) Strength is evaluated by the cube compressive strength of concrete at 28 days and strength ratio *t* at 28 days. The compressive strength of C30 SSAWC should be more than 30 MPa, and the strength ratio *t* of SSAWC should be more than 0.7 according to the code [34].

(4) Expansion of the SSAWC in the curing stage is helpful to completely filling the gap between core members and reinforcing material. Restrained expansion rate at 14 days was applied to evaluate the expansion. Restrained expansion rate at 14 days of SSAWC should be more than 0.03% according to the reference [35].

### 2.2. Main Materials

The materials used in this test were obtained from Xiezhu building materials Co., Ltd., Fuzhou City, China. 

**Figure 1:**
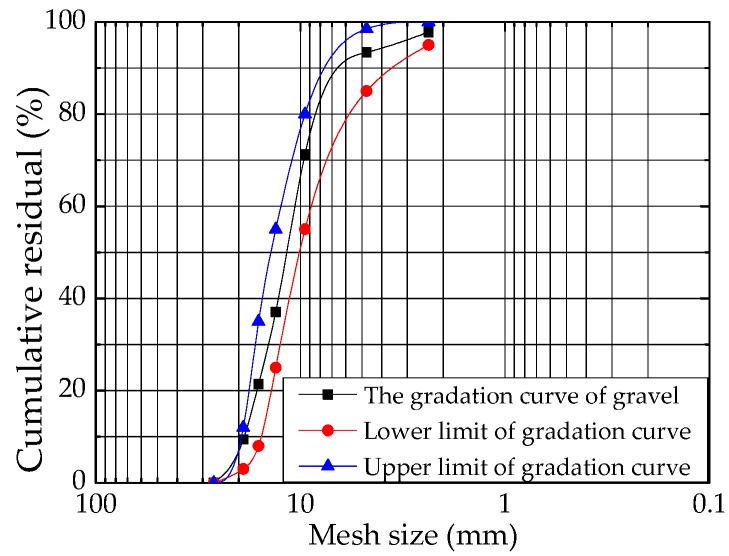
The gradation curve of gravel.

(1) Cement: the most important component of SSAWC, bearing the greatest impact on the strength of SSAWC. The 42.5 N normal Portland cement from China United Cement Corporation, (Beijing, China) was chosen; this kind of cement is used to test the quality of admixtures, which is beneficial to the compatibility test of admixture in this paper. The chemical compositions and physical properties of cement are presented in Table 1 and Table 2, respectively.

(2) Coarse aggregate: the strength and size of coarse aggregate affect both the strength and uniformity of concrete. This is because when the concrete was being poured in, the aggregate sank fast as the size increased, and the concrete became more uneven. To ensure high strength and uniformity of concrete, 5–20 mm of continuously graded tuff gravel was used. The density, crushed index, needle content, and sediment content of the gravels were 2.65 t/m^3^, 7.3%, 2%, and 0.2%, respectively. The gradation curve of the coarse aggregate is shown in Figure 1.

(3) Fine aggregate: the fineness modulus and mud content of fine aggregate related with the water consumption of concrete can also affect the strength and fluidity of SSAWC. Thus, good grade Fujian River sand was chosen and used in this test. The fineness modulus, sediment content, and density of the sand were 2.89, 0.6%, and 2.64 t/m^3^, respectively.

(4) AWA can increase the viscosity and decrease the segregation trend underwater. The type and amount of AWA have the greatest impact on the anti-dispersity. A fiber-type AWA named SBT-NDA, produced by the Jiangsu Soubotte Company (Nanjing, China), was used in this experiment. The recommended dosage is 3% of cement content. The AWC was made conforming to the code [34] and its performance was tested to verify that AWA met the code [34] requirements. The mix ratio of AWC is shown in Table 3, and the performance of AWC shown in Table 4. 

(5) Expansive agent: volume expansion and prestressing in SSAWC are produced by crystallization from the reactions between expansive agent, cement and water [36]. In this test, the HME-III low alkali concrete expansive agent, which is a type-K (ettringite) expansive agent, and a mix of calcium sulfoaluminate and calcium was supplied by the Jiangsu Soubotte Company (Nanjing, China). Recommended addition is 10% of cement content replacing cement. Properties of expansive agent need to be verified by test. The expansive cement mortar was made according to the mix ratio shown in Table 5, and its performance was tested to verify that expansive agent meets the requirements [35], as shown in Table 6.

**Figure 2:**
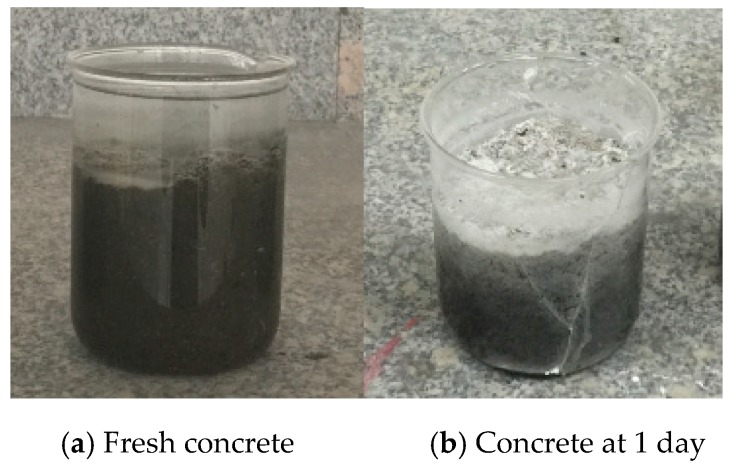
Compatibility test between AWA and expansive agent.

However, we note that the compatibility of expansive agent with AWA becomes a major issue in SSAWC preparation. The flocculation of AWA takes effect before the initial setting of concrete, while the expansion of expansive agent takes effect after the initial setting. The action time of AWA and expansive agent does not conflict with each other in the experiment, that is, they are fully compatible. Moreover, the test of adding expansive agent and AWA to concrete is shown in Figure 2. The Figure 2a shows that the AWA takes effect before the initial setting, and makes the water clear; Figure 2b shows that the expansion agent affects the hardening of the concrete and causes the glass to break at day 1. This implies that the effect of expansive agent and AWA is compatible with each other.

(6) Water-reducing agent: it was selected according to the compatibility of AWA and expansive agent. Research from Khayat and Kamal Henri [20] showed that the fiber-series AWA and naphthalene-series water-reducing agent can be gelatinized, and as a result, the fluidity no longer meets the requirements. The results of Miguel José Oliveira’s experiment [37] implied that a type-K expansive agent is compatible with polycarboxylate superplasticizer. Therefore, polycarboxylate superplasticizer was employed in this test. The recommended dosage is 1% of cement content. The concrete was made according to the mix ratio shown in Table 7, and the performance was tested and compared to verify its role of water reducer. The properties of the polycarboxylate superplasticizer are shown in Table 8. 

(7) Fly ash: it is beneficial to the improvement of the fluidity of fresh AWC and the reduction of the loss of fluidity, but it may be harmful to the strength and anti-dispersity. Meanwhile, the silica fume is good for strength, but harmful to the fluidity. Thus, the fly ash and silica fume are not used in this test in order to ensure the strength of SSAWC.

### 2.3. Mix Design

The concrete mix ratio in this test was designed according to Chinese Codes [34,38]. The relationships between the *f*_cu,0_, *f*_cu,k1_, and *f*_cu,k2_ (the definition of notations in Section 2.3 are shown in Table 9) are shown in Equations (1) and (2):

(1) $$f_{\mathrm{cu},k1}=f_{\mathrm{cu},k2}/t$$

(2) $$f_{\mathrm{cu},0}=f_{\mathrm{cu},k1}+1.645\sigma$$

The calculations of the *w*/*b*, *m*_wo,_ *β*_s_ are given as follows.

(3) $$w/b=m_{w0}/m_{b0}=f_{b}/\left(f_{\mathrm{cu},0}+\alpha_{a}\alpha_{b}f_{b}\right)$$

(4) $$m_{w0}=m_{w0^{\prime }}(1-\beta )$$

(5) $$\beta_{s}=\left(m_{s0}\times 100\%\right)/\left(m_{s0}+m_{g0}\right)$$

(6) $$m_{c0}/\rho_{c}+m_{g0}/\rho_{g}+m_{s0}/\rho_{s}+m_{w0}/\rho_{w}+\alpha =1$$

The mix ratio of SSAWC can be designed according to the procedure as follows.

Step 1: Target compounding strength in practical test. The designed concrete strength of old bridges built decades ago is usually around C30. Thus, the designed strength of SSAWC is set to 30 MPa, namely *f*_cu,k2_ = 30 MPa. *σ* is 5 according to Code [38]. The value of strength ratio *t* is set to 0.7 to ensure the sufficient strength of SSAWC in test. Then the target compounding strength of SSAWC in test can be calculated according to Equations (1) and (2), and *f*_cu,0_ = 30/0.7 + 1.645 × 5 = 51.08 MPa. 

Step 2: Water–binder ratio. According to Code [38], *α*_a_ and *α*_b_ are set to 0.53 and 0.2, respectively. The value of *f*_b_ is 46.75MPa after testing. Then, according to Equation (3), *w*/*b* = 0.53 × 42.5 × 1.1/(51.08 + 0.53 × 0.2 × 42.5 × 1.1) = 0.44. In order to ensure the strength of SSAWC, the actual *w*/*b* is set to 0.43 according to experience. Since fly ash and fume are not used, *w*/*b* = *w*/*c* = 0.43.

Step 3: Water and cement content. Water content can be calculated according to the code [38], i.e., *m*_w0’_ = 258.75 kg/m^3^. If a water-reducing agent is used, the water content can be calculated according to Equation (4). In order to ensure the fluidity of SSAWC, the water reducing rate is set to 20%. So *m*_w0’_ = 258.75 × (1 − 20%) = 207 kg/m^3^, *m*_c0_ = 207/0.43 = 481.4 kg/m^3^. 

Step 4: Sand ratio and aggregate content. The sand ratio can be calculated according to the code [38], i.e., *β*_s_ = 41%. *α* is set to 4 according to properties of water-reducing agent. Aggregate content can be calculated by Equation (5) and Equation (6): *m*_s0_ = 692 kg/m^3^ and *m*_g0_ = 995 kg/m^3^, respectively. 

Step 5: chemical admixture content. It can be determined by the recommended amount of admixture. The content of AWA is 3% of cement content. The content of expansive agent is 10% of cement content. The content of water-reducing agent is 1% of cement content.

To sum up, the basic mix ratio of SSAWC is shown in Table 10.

### 2.4. Specimens Preparation

**Figure 3:**
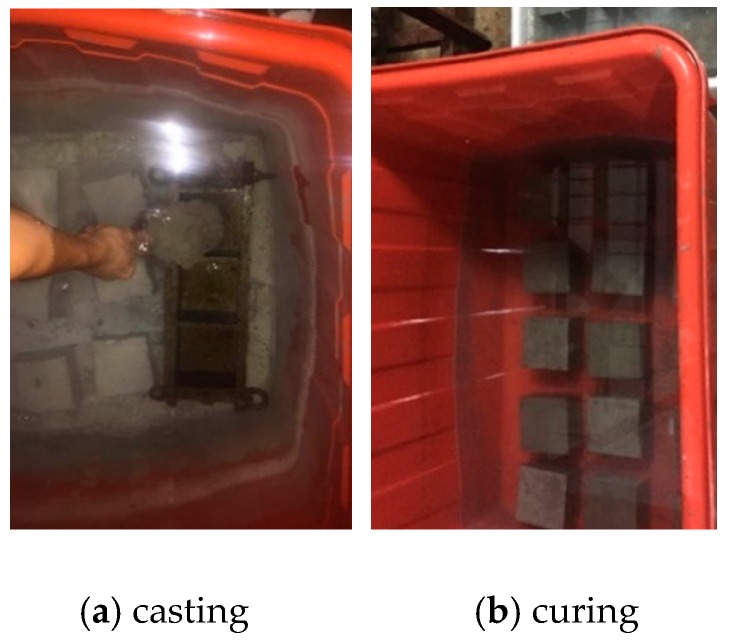
Production process of specimens.

The preparation process of SSAWC specimens is similar to that of normal concrete mixtures (see also Figure 3).

*Step 1: Mixing.* All batches were mixed by an open pan mixer following the same procedure, i.e., (1) the mixer was washed and mixed with a small amount of concrete with the same ratio first so as to make the mixer wall wetted with cement slurry; (2) coarse aggregate, sand, cement, water and chemical admixture were poured into the mixer and mixed for 3 min; (3) wait for 1 min before the mixing was resumed for another two or three minutes; (4) the (2) and (3) were repeated 3 times; (5) the mixed concrete was poured onto the steel plate and mixed by hand for 2 to 3 times. Finally, the homogeneous mixture of SSAWC was produced. 

*Step 2: Casting*. The dimension of the moulds used for specimens forming was 100 mm × 100 mm × 100 mm. A plastic bucket with a height of 450 mm filled with water was used to simulate underwater environment. The cube moulds were placed in water and the distance between the surface of water and the top surface of each mould was approximately 150 mm. Then the SSAWC mixture was poured into the moulds. The casting continued until the moulds were filled up with SSAWC. The specimens were removed from the water and left in air for 10 min after casting to allow for self-leveling and self-compacting of concrete, and then put into the water again.

*Step 3: Curing.* Moulds were removed off all specimens after 2 days of casting, and then the specimens were cured for 28 days in water where the temperature was kept at (20 ± 3) °C.

### 2.5. Test Methods

**Figure 4:**
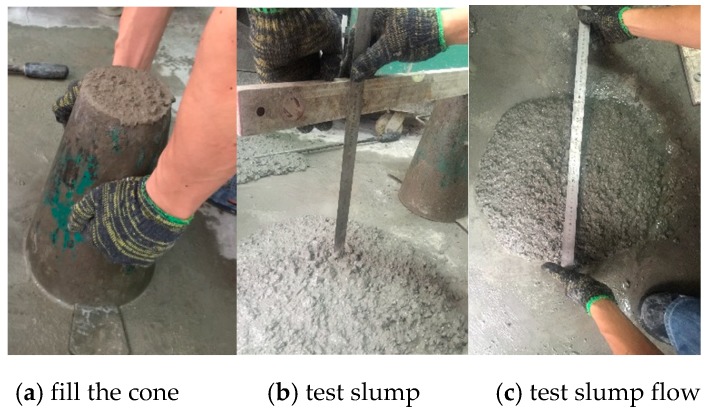
Test of fluidty.

**Figure 5:**
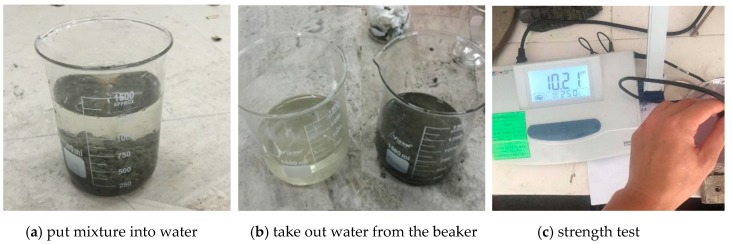
Test of anti-dispersity.

**Figure 6:**
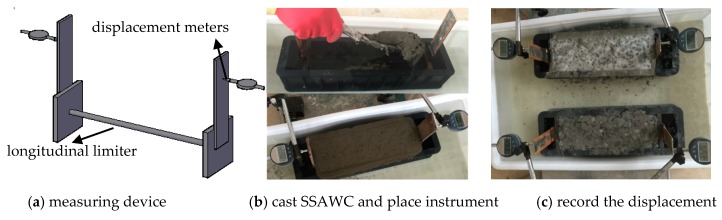
Test of expansive.

(1) Fluidity. Slump and Slump flow were used to evaluate the ease of spreading of SSAWC. Because of high viscosity of SSAWC, the measurement of slump and slump flow was delayed for 2 min after the removal of the cone to allow the flow to take place [20]. As shown in Figure 4, SSAWC was filled into the cone first, and then the cone was removed to allow SSAWC to drop and flow freely; it was only 2 min later that the measurement could be taken; specifically, the height of the SSAWC drop was measured as slump and the diameter of SSAWC was measured as slump flow. The measurement precision was about 5 mm. 

(2) Anti-dispersity. It was evaluated via a pH test as shown in Figure 5. The test contains three parts: (1) 50 g of fresh SSAWC mixture was slowly put into beaker A filled with 800 mL of water at end of mixing, and then stood for 3 mins; (2) 600 mL of water only (with no concrete) was drawn into beaker B from beaker A with a straw in 1 min; (3) the pH value of water in beaker B was measured at once with a PHS-3E pH meter produced by Shanghai INESA Scientific Instrument Co., Ltd. (Shanghai, China).

(3) Strength. The cube compressive strength of concrete at 28 days was measured and taken for the strength of concrete. In this test, the specimen was aligned to the center of the pressure plates. The load was uniformly applied on the top surface of the specimen at a controlled speed of 0.2 N/mm^2^ to 0.3 N/mm^2^ per second until the specimen was damaged. 

(4) Expansibility. The restrained expansion rate at 14 days was used to evaluate expansibility of SSAWC. As shown in Figure 6a, the restrained expansion rate of each specimen was measured by two displacement meters placed on both ends of the specimen. The method of measuring restrained expansion rate consisted of 3 steps (see Figure 6): (1) a cube mould with a dimension of 100 mm × 100 mm × 400 mm was placed in the water bucket; and then SSAWC was casted into the mould; (2) the top surface of SSAWC was smoothed and mounted with the displacement meters; (3) the displacement was measured and recorded every 24 h.

The performances of SSAWC produced by the basic mix ratio is shown in Table 11. We can see that the fluidity, anti-dispersity, strength and expansibility of SSAWC produced by the basic mix ratio in Table 10 all meet the requirements outlined in Section 2.1. It shows that the mix ratio design method of normal concrete [38] is suitable for SSAWC. However, the slump flow of SSAWC can only meets the minimum requirements described in Section 2.1, while the anti-dispersity and strength are superior to the required values. If the mix ratio helps reduce the amount of AWA and cement, the slump flow would be increased and the cost would be reduced [15]. Therefore, it is necessary to optimize the mix ratio of SSAWC. 

### 2.6. Orthogonal Test Design

The orthogonal test design method was used to optimize the mix ratio of SSAWC. It is not only a rapid and economical experiment design method, but also an important scheme for multifactor and multilevel researches so far [39,40]. In general, practical engineering experiment design usually involves three or more influential parameters, and requires full factorial design analysis. In this test, the minimum number of trials would be 3^5^ = 243 for full factorial experiments with five influential parameters (factors) and three levels. Obviously, it is difficult and expensive for researchers to carry out such a large number of repeated experiments. As an alternative, the orthogonal test design method selects uniformly distributed key points in the test range to adequately represent the overall situation of the full factorial experiments [41].

In this test design, five factors known as the main factors affecting concrete performances were selected as the design factors, namely water–cement ratio, AWA, expansive agent, water-reducing agent, and sand rate. Each factor contained three levels as shown in Table 12. Thus, there were a total of eighteen test groups and the orthogonal array used in the design is described as L18 (35), where L is the symbol of orthogonal design; 18 is the number of experiments; three is the number of factor levels, and five is the number of factors. The list of the L18 (35) orthogonal levels is shown in Table 13.

## 3. Results and Discussion

The results of the orthogonal test are shown in Table 14. The range analysis was carried out to measure the significance order of influence of each factor on the test results. In fact, the range analysis assumes that the influence of each factor on the results is balanced, and the level difference of a single factor is caused by the factors themselves.

In the range analysis, *R_j_* represents the range, which means the difference between the maximum and minimum average values under the level of factors in the *j* column; and the corresponding computation formula is as follows.

*R_j_* = max (*k_1j_, k_2j_; …; k_ij_*) − min (*k_1j_; k_2j_; …; k_ij_*) (7)

where, *k_ij_* represents the total of corresponding test data of Level *i* of the factors in the *j* column. The larger *R_j_* is, the more greatly the variation of factor affects the measured results; that is, this factor has greater impact on the measured results [42]. In this study, the value range of *i* is from 1 to 3, and the value range of *j* is from A to E.

### 3.1. Fluidity

#### 3.1.1. Range Analysis for Fluidity Test

The test results of slump of each mix ratio and process of range analysis is listed in Table 15. *k_ij_* represents the total of testing results under Level *i* of *j* factor, and the optimal level is defined as the maximum or minimum of *k_ij_* for each column corresponding to the factor. *K_ij_* is the average value of *k_ij_*. For example, *K*_1A_ = [∑ (slump*_group1_* + slump*_group2_* + slump*_group3_* + slump*_group10_* + slump*_group11_* + slump*_group12_*)]/6 = (230 + 235 + 225 + 230 + 235 + 230)/6 = 230.8 and *R*_A_ = *K*_3A_ − *K*_1A_ = 243.3 − 230.8 = 12.5. Likewise, the range analysis for the slump flow test is shown in Table 16. 

Based on the range analysis, the order of significance of the above factors for the slump is as follows; A (*w*/*b*) > B (AWA) > E (sand rate) > C (expansive agent) > D (water-reducing agent). Thus, the optimal combination of factors for slump is A_3_B_1_C_2_D_2_E_2_. Likewise, the order of significance of the above factors for the slump flow is listed as follows; A (*w*/*b*) > B (AWA) > E (sand rate) > C (expansive agent) > D (water-reducing agent). Thus, the optimal combination of factors for slump flow is A_3_B_1_C_1_D_3_E_2_.

#### 3.1.2. Effect of Factors on Fluidity

From Table 14, it can be seen that the slump of all groups of SSAWC meets the requirements in Section 2.1, while the slump flow of 10 groups of SSAWC fails to meet the requirement. This is due to the low *w*/*b* or high AWA content or both. The results of range analysis show that the *w*/*b* has the greatest effect on fluidity of SSAWC and the fluidity increases with the increment of water–cement ratio, this is similar to AWC [43]. Consistent with Heniegal [19] and Zhang’s [23] research results, the effect of AWA on fluidity ranks the second place, and the fluidity decreases with the increase of AWA. The effect of AWA is more obvious on slump flow than on slump, because the effect of flocculation produced by AWA has greater impact on the horizontal flow diffusion of concrete than the vertical collapse. The effect of sand rate on fluidity ranks third place. The influence curve of sand ratio on fluidity takes a shape of parabola. In other words, the fluidity of SSAWC increases with the increase of sand rate, but the sand rate over 41% on the other hand can compromise the improvement remarkably. This result of the test is similar to that of Zhang [22]. The expansive agent and the water-reducing agent have less effect on the fluidity compared with other factors, although the fluidity of SSAWC increases with the increase of water-reducing agent and the decrease of expansive agent. This is because the underwater environment weakens the effect of the expansive agent and the water-reducing agent on the fluidity. 

### 3.2. Expansibility

#### 3.2.1. Range Analysis for Expansibility Test

The range analysis for expansibility test is shown in Table 17. Based on the range analysis, the order of significance of the above factors for limited expansive agents: C (expansive agent) > B (AWA) > A (*w*/*b*) > E (sand rate) > D (water-reducing agent). Thus, the optimal combination of factors for expansibility is A_1_B_2_C_3_D_3_E_3_.

#### 3.2.2. Effect of Factors on Expansibility

It is shown in Table 14 that restrained expansion rate at 14 days of four groups of SSAWC failed to meet the requirement in Section 2.1 due to low expansive agent content. From Table 17, it is clear that the content of expansive admixture has the greatest effect on expansibility of SSAWC, and the relationship of expansive admixture and limited expansion ratio is positively correlated. This is consistent with the test results of Nagataki [35]. The effect of AWA on expansibility ranks the second place, and it increases with the addition of AWA, but the trend reverses when the addition is more than 3%. This is because the concrete can achieve good performance with an appropriate amount of AWA, like cohesion, water retention, and fluidity, the then concrete becomes denser and leads to a large restrained expansion rate. This result of the test is similar to that of Lu [44]. Compared with the AWA, the *w*/*b* has less effect on expansibility, and it decreases with the increase of water–cement ratio, because large interstices in the concrete can absorb the expansion energy and reduce the restrained expansion rate. On the other hand, the influence of water-reducing agent and sand rate on expansibility is insignificant.

### 3.3. Anti-Dispersity

#### 3.3.1. Range Analysis for Anti-Dispersity Test

The range analysis results for anti-dispersity test are shown in Table 18. Based on the range analysis, the order of significance of the above factors for anti-dispersity is B (AWA) > A (*w*/*b*) > D (water-reducing agent) > E (sand rate) > C (expansive agent). Thus, the optimal combination of factors for anti-dispersity is A_1_B_3_C_2_D_1_E_3_.

#### 3.3.2. Effect of Factors on Anti-Dispersity

From Table 14, it is found that the pH values of all groups of SSAWC meet the requirement in Section 2.1. The results indicate that when the content of SBT-NDA AWA reaches 2.5% of the cement content, the SSAWC still has good anti-dispersity. From Table 18, it is seen that the content of AWA has the greatest influence on anti-dispersity of SSAWC, and the relationship of AWA and anti-dispersity is positively correlated, similar to AWC [15]. The effect of *w*/*b* on anti-dispersity is in second place, which decreases with the increasing water–cement ratio. This is because a large *w*/*b* increases water consumption and water content in the interstices, then the surface tension decreases and the cohesion becomes poor [20]. The effect of water-reducing agent on the pH value is similar to the *w*/*b*, but the effect was reduced when the content exceeds the 1%. This is segregation of the SSAWC caused by high content of superplasticizer, which would eventually affect the flocculation of AWA. Expansive agent and sand rate have less effect on anti-dispersity.

### 3.4. Strength

#### 3.4.1. Range Analysis for Strength Test

The range analysis results for underwater intensity test are shown in Table 19. The order of significance of the above factors for strength is A (*w*/*b*) > C (expansive agent) > B (AWA) > D (water-reducing agent) > E (sand rate). Thus, the optimal combination of factors for underwater strength is A_1_B_2_C_2_D_1_E_2_.

#### 3.4.2. Effect of Factors on Strength

It is shown in Table 14 that the compressive strength and compressive strength ratio *t* of all groups of SSAWC meet the requirement in Section 2.1. From Table 19, it is found that the *w*/*b* has the greatest influence on the strength of SSAWC, but the *w*/*b* is negative correlation with the strength, just like normal concrete [38]. The influence of the AWA, expansive agent, and water-reducing agent on strength is similar. Specifically, the strength of SSAWC can be enhanced with the addition of each of the above agents in the initial stage. However, when the admixture content exceeds a certain value (the content of AWA, expansive agent and water-reducing agent is 3%, 10%, and 0.8%, respectively), the strength will decrease. This is because excessive dosage of anti-dispersant agent will reduce the fluidity significantly, and the excessive dosage of the expansion agent in place of cement will reduce the amount of cement slurry [27]; the excessive dosage of the water-reducing agent will make concrete bleed. From the table 19, we can also see that the sand rate has the least effect on the underwater strength.

### 3.5. Determining the Optimal Mix Ratio

#### 3.5.1. Optical Mix Ratio

From the above-mentioned, we can see that there can be various combinations of factors. Therefore, the optimal mix ratio of SSAWC should be determined for individual factors. 

(1) Water–cement ratio. Water–cement ratio has the greatest salutary influence on strength, and is negatively correlated with strength. However, it has a large adverse impact on the fluidity. From the test results in Table 14, it can be found that the slump flow of 10 groups failed to meet the target requirements in Section 2.1, but the strength of every group is greater than the target value. In order to increase the fluidity, it is appropriate to increase the water–cement ratio. As a consequence, A_2_ (*w*/*c* = 0.43) was selected as the optimal level of water–cement ratio.

(2) AWA. AWA has the greatest effect on anti-dispersity, but excessive dosage is not beneficial to the fluidity and strength. From the test results shown in Table 14, it is found that the anti-dispersity of each group meets the target requirement. Therefore, B_1_ (2.5% of cement content) was chosen as the optimal level to save cost. 

(3) The expansive agent. It has the greatest effect on expansibility, and is positively correlated with the restrained expansion rate, but excessive addition of expansive agent will reduce the intensity and the fluidity. As shown in Table 14, the restrained expansion rate of four groups failed to meet the target requirement when the amount of expansive agent is 7% of the cement content. Hence, C_2_ (10% of cement content) was picked as the optimal level. 

(4) Water-reducing agent. It is beneficial to fluidity and strength, but when the amount is greater than 1% of cement in mass, the impact is not obvious. Therefore, D_2_ (1% of cement content) was taken for the optimal level. 

(5) Sand rate. It is helpful to the enhancement of fluidity and anti-dispersity, but when the value of sand rate is greater than 41%, the impact of sand ratio on the fluidity and anti-dispersity will become negative. In order to ensure the fluidity of concrete, E_2_ (*β*_s_=41%) was chosen as the optimal level. 

Therefore, the C30 SSAWC optimal mix ratio is A_2_B_1_C_2_D_2_E_2_, as shown in Table 20. The water–cement ratio is 0.43, AWA dosage is 2.5%, expansive agent amount is 10%, sand rate is 41%, and water-reducing agent is 1%, respectively. 

#### 3.5.2. Validation of Optimal Mix Ratio

One group of specimens with seven test-pieces was used to validate the proposed optimal mix ratio. The performance of SSAWC using the optimal mix ratio is shown in Table 21. We can see that its performances can meet the requirements in Section 2.1. This indicates that SSAWC is feasible and applicable to strengthening methods without drainage. 

### 3.6. Mix Ratio of SSAWC in Other Strength Grades

Mix ratio of SSAWC in other strength grades was developed as follows. (1) The basic mix ratio was calculated according to the method in Section 2.3; (2) the relevant indexes were adjusted according to the performances of SSAWC with basic mix ratio; (3) *w*/*b* was adjusted to change the strength of SSAWC, the content of AWA and sand rate were adjusted to change the fluidity of SSAWC; (4) the content of expansive agent was adjusted to change the expansibility of SSAWC; and (5) the water consumption of SSAWC was adjusted according to the water content of aggregate and construction environment.

## 4. Conclusions

Based on the studies of AWC and SSC, a series of experiments and analyses have been carried out in this study to propose the optimal mix ratio of SSAWC used in nondrainage strengthening, and reveal the effect of the designed factors on its performance, and increase the bond strength. The following conclusions have been drawn.

(1) The mix ratio design of SSWAC can be carried out according to the method designed for normal concrete, but appropriate adjustments should be made. 

(2) The performance of SSAWC with strength level of C30 can obtain ideal performance with the following mix ratio, water cement–ratio 0.43, the reasonable value range of AWA agent 2.5%, the reasonable value of expansive agent 10%, sand rate 41%, and water-reducing agent 1%. The mix ratio of SSAWC in other strength grade can be developed using the same method as described in this paper. 

(3) The expansibility and self-stress of SSAWC are obvious to observe, and the restrained expansion ratio at 14 days can reach 0.027–0.053%. In a word, SSAWC is endowed with the functions of expansion and self-stress production. 

(4) The expansive agent has a large influence on the strength of SSAWC. The compressive strength ratio *t* is increased compared with AWC. The maximal value of the compressive strength ratio is 0.92, close to the peak ever measured in existing researches. The expansive agent is beneficial to the fluidity of SSAWC, but the effect turns negative if the content of expansive agent exceeds a certain value (10% of cement content).

(5) The expansive agent and AWA agent used in the test are compatible. The effect of expansive agent on anti-dispersity is insignificant. 

The above-mentioned conclusions are drawn from limited experimental data, and more experiments will be conducted in the future with SSAWC of other strength grades. In addition, the durability and the performance of SSAWC in no-drainage strengthening will also be further studied.

## Figures and Tables

**Table 1:** Chemical compositions of cement.

Chemical Composition	SiO_2_	Al_2_O_3_	Fe_2_O_3_	CaO	MgO	SO_3_	Na_2_Oeq
Results by wt. (%)	21.88	4.49	3.45	64.65	2.36	2.44	0.51

**Table 2:** Properties of cement.

Water Requirement of Normal Consistency (%)	Setting Time (min)		Cube Compressive Strength (MPa)		Flexural Strength (MPa)		Volume Stability	Density (g/cm^3^)
	Initial Time	Final Time	3d	28d	3d	28d		
25.2	138	218	28.9	49.2	6.2	8.9	Qualified *	3.16

*: The quality of volume stability indicates that the volume of cement varies uniformly during the hardening process, which conforms to the requirements of the specification.

**Table 3:** The mix ratio of AWC.

Cement (kg/m^3^)	*w*/*b* *^1^	AWA (Percent Cement)	Sand (kg/m^3^)	Stone (kg/m^3^)	*β*_s_ *^2^
360	0.667	3	664	996	0.6

*^1^: *w*/*b*: water-binder ratio, wherein mineral admixtures are not used, thus *w*/*b* = *w*/*c*. *^2^: *β*_s_: sand rate, the ratio of the quality of fine aggregate to the quality of all of aggregate.

**Table 4:** Properties of anti-washout underwater concrete (AWC).

Bleeding Rate *^1^ (%)	Gas Content (%)	pH	Setting Time (h)	
			Initial Time	Final Time
0	3.4	10.9	18.3	21

*^1^: Bleeding rate: the ratio of bleeding water to water consumption of concrete.

**Table 5:** The mix ratio of expansive cement mortar.

Cement (kg/m^3^)	*w*/*b*	Expansive Agent (Percent Cement)	Sand (kg/m^3^)
405	0.5	3	1350

**Table 6:** Properties of expansive cement mortar.

Restrained Expansion Rate after 7 Days *^1^ (%)	Setting Time (min)		Cube Compressive Strength (MPa)		Fineness
	Initial Time	Final Time	7d	28d	Specific Surface Area (m³/kg)
0.032	175	310	24.8	47.8	403

*^1^: Test of restrained expansion rate after 7 days is described in Section 2.5.

**Table 7:** The mix ratio of concrete.

Concrete	Cement (kg/m^3^)	*w*/*b*	Water-Reducing Agent (Percent Cement)	Sand (kg/m^3^)	Stone (kg/m^3^)	*β*_s_
M1	360	0.667	0	715	945	0.43
M2	360	0.5	1	715	945	0.43

**Table 8:** Properties of polycarboxylate superplasticizer.

Water Reducing Ratio *^1^ (%)	Ratio of Bleeding Rate *^2^ (%)	Setting Time Different *^3^ (min)		Cube Compressive Strength Ratio *^4^ (%)		Gas Content (%)
		Initial Time	Final Time	7d	28d	
25	54.7	+100	+120	158	138	4

*^1^: Water reducing ratio means that the ratio of different water consumption between M1 and M2 to water consumption of M1 when the slump was the same. *^2^: Ratio of bleeding rate: M2 bleeding rate/M1 bleeding rate. *^3^: Setting time different: setting time of M2 and setting time of M1. *^4^: Compressive strength ratio: compressive strength of M2/compressive strength of M1.

**Table 9:** List of notations.

Notations	Definition
*f*_cu,0_	target compounding strength of SSAWC in test
*f*_cu,k1_	design strengths of SSAWC casted and cured in air
*f*_cu,k2_	design strengths of SSAWC casted and cured underwater
*f*_b_	the compressive strength of the cementing material at 28 days
*t*	compressive strength ratio of specimens made underwater to those made in air
*σ*	standard deviation of strength
*w*/*b*, *w*/*c*, *#x3B2;*	water–binder ratio, water–cement ratio, and water–reducing ratio, respectively
*m*_c0_, *m*_b0_	the masses of cement and binder in 1 m^3^ of SSAWC, respectively
*m*_g0_, *m*_s0_, *m*_w0_	the masses of gravel, sand, and water in 1 m^3^ of SSAWC, respectively
*m*_wo’_	the water consumptions before using water-reducing agent in 1 m^3^ of SSAWC
*α*_a_, *α*_b_	regression coefficient
*ρ*_c_, *ρ*_g_, *ρ*_s_, *ρ*_w_	the densities of cement, fume, gravel, sand, and water, respectively
*α*	the volume of air in per unit volume of SSAWC
*β*_s_	the ratio of the quality of fine aggregate to the quality of all of aggregate

**Table 10:** The basic mix ratio of SSAWC.

Cement (kg/m^3^)	*w*/*b*	Admixture (Percent Cement) *			Aggregate (kg/m^3^)		*β*_s_ (%)
		AWA	EA	WRA	Sand	Stone	
433.3	0.43	3	10	1	692	995	41

*: EA = expansive agent, WRA = water-reducing agent.

**Table 11:** Properties of self-stressed anti-washout underwater concrete (SSAWC) with basic mix ratio.

Slump (mm)	Slump Flow (mm)	Restrained Expansion Rate at 14 Days (%)	pH Value	Underwater Strength at 28 Days (MPa)	Strength at 28 Days (MPa)	Strength Ratio
250	420	0.038	10.22	37.2	45.9	0.810

**Table 12:** The values of different levels of the factors in orthogonal test.

Factors	A: *w*/*b*	B: AWA (Percent Cement)	C: EA (Percent Cement)	D: WRA (Percent Cement)	E: *β*_s_ (%)
Level 1	0.41	2.5	7	0.8	38
Level 2	0.43	3	10	1	41
Level 3	0.45	3.5	13	1.2	44

**Table 13:** The mix ratio of orthogonal tests.

Group	Cement (kg/m^3^)	*w*/*b*	Admixture (Percent Cement)			Aggregate (kg/m^3^)		*β*_s_ (%)	Mix Ratio
			AWA	EA	WRA	Sand	Stone		
1	469.5	0.41	2.5	7	0.8	634	1034	38	A_1_B_1_C_1_D_1_E_1_ *
2	454.4	0.41	3	10	1	684	984	41	A_1_B_2_C_2_D_2_E_2_
3	439.2	0.41	3.5	13	1.2	717	950	44	A_1_B_3_C_3_D_3_E_3_
4	447.7	0.43	2.5	7	1	692	995	41	A_2_B_1_C_1_D_2_E_2_
5	433.3	0.43	3	10	1.2	742	945	44	A_2_B_2_C_2_D_3_E_3_
6	418.8	0.43	3.5	13	0.8	641	1046	38	A_2_B_3_C_3_D_1_E_1_
7	414	0.45	2.5	10	0.8	750	955	44	A_3_B_1_C_2_D_1_E_3_
8	400.2	0.45	3	13	1	648	1057	38	A_3_B_2_C_3_D_2_E_1_
9	427.8	0.45	3.5	7	1.2	699	1006	41	A_3_B_3_C_1_D_3_E_2_
10	439.2	0.41	2.5	13	1.2	684	984	41	A_1_B_1_C_3_D_3_E_2_
11	469.5	0.41	3	7	0.8	734	934	44	A_1_B_2_C_1_D_1_E_3_
12	454.4	0.41	3.5	10	1	634	1034	38	A_1_B_3_C_2_D_2_E_1_
13	433.3	0.43	2.5	10	1.2	641	1046	38	A_2_B_1_C_2_D_3_E_1_
14	418.8	0.43	3	13	0.8	692	995	41	A_2_B_2_C_3_D_1_E_2_
15	447.7	0.43	3.5	7	1	742	945	44	A_2_B_3_C_1_D_2_E_3_
16	400.2	0.45	2.5	13	1	750	955	44	A_3_B_1_C_3_D_2_E_3_
17	427.8	0.45	3	7	1.2	648	1057	38	A_3_B_2_C_1_D_3_E_1_
18	414	0.45	3.5	10	0.8	699	1006	41	A_3_B_3_C_2_D_1_E_2_

*: The subscript “1” means that the contents of the five factors were set to the values of the first levels as shown in Table 13.

**Table 14:** Orthogonal test results.

Group	Slump (mm)	Slump Flow (mm)	Restrained Expansion Rate at 14 Days (%)	pH Value	Strength at 28 Days (MPa)	Underwater Strength at 28 Days (MPa)	Strength Ratio
1	230	415	0.027	11.05	47.4	38.5	0.813
2	235	400	0.042	10.22	48.5	39.4	0.813
3	225	395	0.053	9.32	48.0	37.3	0.778
4	245	430	0.028	11.20	38.9	35.7	0.917
5	245	420	0.040	10.27	45.6	36.7	0.806
6	235	395	0.048	9.57	46.0	36.2	0.787
7	250	425	0.035	11.37	43.1	35.6	0.825
8	250	420	0.049	10.54	45.4	35.2	0.774
9	250	430	0.028	9.67	45.2	35.2	0.778
10	230	420	0.050	11.23	41.9	36.0	0.860
11	235	405	0.036	9.87	44.1	36.3	0.823
12	230	395	0.039	9.52	43.5	36.4	0.838
13	225	400	0.035	11.23	44.7	35.5	0.795
14	230	400	0.052	10.22	42.5	36.1	0.850
15	210	395	0.027	9.67	40.8	32.8	0.805
16	245	430	0.047	11.35	43.8	34.6	0.790
17	230	420	0.032	10.63	44.7	35.1	0.786
18	235	415	0.034	9.52	44.4	35.2	0.795

**Table 15:** Range analysis process based on the test results of slump.

Group Number	Mix Ratio	Levels of the Factors					Slump (mm)
		A	B	C	D	E	
1	A_1_B_1_C_1_D_1_E_1_	1	1	1	1	1	230
2	A_1_B_2_C_2_D_2_E_2_	1	2	2	2	2	235
3	A_1_B_3_C_3_D_3_E_3_	1	3	3	3	3	225
4	A_2_B_1_C_1_D_2_E_2_	2	1	1	2	2	245
5	A_2_B_2_C_2_D_3_E_3_	2	2	2	3	3	245
6	A_2_B_3_C_3_D_1_E_1_	2	3	3	1	1	235
7	A_3_B_1_C_2_D_1_E_3_	3	1	2	1	3	250
8	A_3_B_2_C_3_D_2_E_1_	3	2	3	2	1	250
9	A_3_B_3_C_1_D_3_E_2_	3	3	1	3	2	250
10	A_1_B_1_C_3_D_3_E_2_	1	1	3	3	2	230
11	A_1_B_2_C_1_D_1_E_3_	1	2	1	1	3	235
12	A_1_B_3_C_2_D_2_E_1_	1	3	2	2	1	230
13	A_2_B_1_C_2_D_3_E_1_	2	1	2	3	1	225
14	A_2_B_2_C_3_D_1_E_2_	2	2	3	1	2	230
15	A_2_B_3_C_1_D_2_E_3_	2	3	1	2	3	210
16	A_3_B_1_C_3_D_2_E_3_	3	1	3	2	3	245
17	A_3_B_2_C_1_D_3_E_1_	3	2	1	3	1	230
18	A_3_B_3_C_2_D_1_E_2_	3	3	2	1	2	235
K_1,j_		230.8	237.5	233.3	235.8	233.3	
K_2,j_		231.7	236.7	236.7	235.8	237.5	
K_3,j_		243.3	230.8	235.8	234.2	235.0	
R_j_		12.5	6.7	3.4	1.6	4.2	

**Table 16:** Range analysis of slump flow test results.

Number	A	B	C	D	E
K_1,j_	405.0	420.0	415.0	409.2	409.2
K_2,j_	406.7	410.8	409.2	411.7	415.8
K_3,j_	423.3	404.2	410.0	414.2	411.7
R_j_	18.3	15.8	5.8	5.0	6.6

**Table 17:** Range analysis of expansibility test results.

Number	A	B	C	D	E
K_1,j_	0.041	0.037	0.030	0.039	0.038
K_2,j_	0.038	0.042	0.038	0.039	0.039
K_3,j_	0.038	0.038	0.050	0.040	0.040
R_j_	0.003	0.006	0.020	0.001	0.002

**Table 18:** Range analysis results of anti-dispersity test.

Number	A	B	C	D	E
K_1,j_	10.17	11.24	10.35	10.27	10.42
K_2,j_	10.36	10.26	10.32	10.38	10.31
K_3,j_	10.51	9.55	10.37	10.39	10.31
R_j_	0.34	1.69	0.05	0.12	0.11

**Table 19:** Range analysis of strength test results.

Number	A	B	C	D	E
K_1,j_	37.33	35.92	35.52	36.33	36.17
K_2,j_	35.43	36.48	36.50	35.62	36.18
K_3,j_	35.15	35.53	35.92	35.98	35.57
R_j_	2.18	0.95	0.98	0.71	0.61

**Table 20:** The optimal mix ratio of SSAWC (kg/m^3^).

Cement (kg/m^3^)	*w*/*b*	Admixture (Percent Cement)			Aggregate (kg/m^3^)		*β*_s_ (%)
		AWA	EA	WRA	Sand	Stone	
433.3	0.43	2.5	10	1	692	995	41

**Table 21:** Index for optimal mix ratio of SSAWC.

Slump (mm)	Slump Flow (mm)	Restrained Expansion Rate after 14 Days (%)	pH Value	Underwater Strength at 28 Days (MPa)	Strength at 28 Days (MPa)	Strength Ratio
245	440	0.041	11.20	35.3	42.9	0.823
